# The Determinants of Conspiracy Beliefs Related to the COVID-19 Pandemic in a Nationally Representative Sample of Internet Users

**DOI:** 10.3390/ijerph17217818

**Published:** 2020-10-26

**Authors:** Mariusz Duplaga

**Affiliations:** Department of Health Promotion and e-Health, Institute of Public Health, Faculty of Health Sciences, Jagiellonian University Medical College, Grzegorzecka Str. 20, 31-531 Krakow, Poland; mariusz.duplaga@uj.edu.pl

**Keywords:** COVID-19, pandemic, infodemic, fake news, misinformation, disinformation, conspiracy theory, health literacy, eHealth literacy

## Abstract

An overwhelming flood of misinformation is accompanying the pandemic of COVID-19. Fake news and conspiracy theories are so prevalent that the World Health Organization started as early as February 2020 to use the term “infodemic”. This paper is focused on the assessment of the prevalence of beliefs in conspiracy theories related to COVID-19 in Polish society. The association of support for conspiracy theories with sociodemographic variables, health literacy (HL) and eHealth literacy (eHL) was studied. The analysis reported here was based on the data from an online survey of a representative sample (*n* = 1002) of the adult population of Polish Internet users. The multivariate linear regression for the COVID-19-related conspiracy belief score (CCBS) and logistic regression models for the support of individual conspiracy theories was developed. The percentage of supporters of particular conspiracy theories in the study sample ranged from 43% to 56%. The CCBS was significantly associated with age, education level, vocational status and both HL and eHL. However, it was lower for persons with higher HL (regression coefficient (B) = −0.04, *p* < 0.001) but higher for those with higher eHL (B = 0.04, *p* = 0.038). The most influential predictors of CCBS were age (standardised regression coefficient (β) = −0.21) and education level (β from 0.08 to 0.16 for respondents with lower education levels and those with master’s degrees). In conclusion, younger persons rather than older, those with a lower rather than with a higher level of education, employees rather than students and persons with lower rather than higher HL were more likely to believe the conspiracy theories. Surprisingly, contrary to expectations, higher eHL was significantly associated with greater belief in such theories.

## 1. Introduction

The COVID-19 pandemic, caused by a new coronavirus, has incited a flood of fake news and conspiracy theories. As early as February 2020, the World Health Organization (WHO) announced that the COVID-19 pandemic was being accompanied by an “infodemic” of misinformation [1].

The COVID-19-related infodemic is propelled by various forms of distorted information, taking the form of conspiracy theories or fake news. A vocabulary definition of a conspiracy theory is “a theory that rejects the standard explanation for an event and instead credits a covert group or organisation with carrying out a secret plot” [2]. Conspiracy theories are not only present in the domain of political life. Oliver and Wood observed that at least 49% of the population living in modern societies agree with at least one medical conspiracy and 18% believe three or more [3]. It is disturbing that medical conspiracism is associated with the increasing use of alternative medicine and the rejection of traditional medicine [3]. According to Galliford and Furnham, belief in political conspiracies is strongly positively correlated with belief in medical conspiracies [4]. There is also evidence that many modern health concerns are significantly associated with medical conspiracy theories [5].

The term “fake news” has gained significant popularity, and notoriety, in recent years, but it is not a new occurrence as fake news was being presented in the tabloid press in the early years of the 20th century [6]. Baines and Elliott have remarked that people should differentiate between the various forms of distorted information that can be spread during periods of a public health emergency, such as during the current COVID-19 pandemic [7]. According to Lazer et al. [8], in addition to fake news, it is possible to identify misinformation and disinformation. These authors defined fake news as “information that mimics the output of the news media in form, but not in organisational process or intent”. Fake news emerges when the organisation issuing it does not adhere to the editorial norms and processes which assure the appropriate quality of information. In turn, misinformation is false or misleading information and disinformation is false information which is purposely spread to deceive people. It seems that most emphasis is put on misinformation and it is regarded as unintentional false information. Some authors also use the term “malinformation”, defined as “reconfigured true information” [7,9].

According to the report published by the Reuters Institute, 59% of misinformation originates from reconfigured information, whereas 38% is deliberately fabricated information [9]. Reconfigured misinformation results from the inclusion of misleading or manipulated content, or providing a false context, but fabricated misinformation emerges from totally false content, or, more rarely, imposter content. A small part of misinformation, only about 3%, is intended as satire or parody. 

The infodemic associated with the COVID-19 pandemic is also related to the rapid spread of many conspiracy theories [10]. One of the most popular is one suggesting that the SARS-CoV-2 virus was developed in a laboratory and released, either accidentally or, in extreme versions, deliberately, to spread chaos and to disrupt the economies of many countries and the whole world. Some news propagated on social media even claimed that it is a type of bioweapon [11]. 

One extreme conspiracy theory is one that denies the existence of the COVID-19 pandemic [11,12]. It suggests that preventive action undertaken to fight the pandemic by specific countries is actually intended to achieve political aims or conceal other problems [13]. Surprisingly, there is a significant group of believers of such a theory. 

In most countries, tracing the contacts of infected persons was based on various information technologies, including mobile applications [14]. The degree of interference with privacy varied significantly and depended on the applied solutions; it ranged from the mandatory use of a mobile application through tracing potential contacts based on transactions made with credit cards and to the use of already installed video surveillance systems. Consequently, concerns about intrusion into individual privacy have taken the form of claims that particular governments will use such systems after the cessation of the pandemic to monitor the population for political purposes [15].

Spreading conspiracy theories and misinformation may lead to many people in society rejecting rational measures known to reduce the risk of infection and combatting the pandemic. For many people, identifying the scientifically justified recommendations in the flood of fake news and misinformation will be a challenge. During the current pandemic, the proposed methods of preventing the infection, such as drinking alcohol, spraying alcohol or chlorine all over the body, eating garlic, taking hot baths and the use of hand dryers, were spread by social media [16]. According to some reports, the number of Iranians admitted to hospitals with methanol intoxication increased significantly during the COVID-19 pandemic, probably due to myths spread on social media falsely promoting the protective effect of alcohol consumption [17]. Apart from the popularisation of unproven and unlikely preventative measures, misinformation may also result in individuals questioning, or ignoring, the recommendations issued by medical organisations and authorities [18,19]. People who are mis- or disinformed are less likely to adhere to the COVID-19-related guidelines [20]. According to Teovanovic et al., of all the analysed irrational beliefs, those related to COVID-19 have had the most detrimental effect on the related health behaviours [21]. Another team showed that a person’s rejection of COVID-19-related conspiracy theories was associated with their compliance with recommended social distancing [22]. Some authors reported that misplaced health protective measures and help-seeking behaviours, as a consequence of media exposure, has led to overburdening of some health care facilities and overuse of the available resources [23]. Interestingly, it seems that a longer duration of the pandemic is associated with a greater level of antisocial behaviours, at least according to the media coverage [24].

The WHO defines health literacy (HL) as “the cognitive and social skills which determine the motivation and ability of individuals to gain access, understand and use information in ways which promote and maintain good health” [25]. The European Health Literacy Project (HLS-EU) proposed an integrated model of HL relating key activities on health-related information to three domains: health promotion, disease prevention and health care [26]. The provision of evidence-based information and support for the health literacy (HL) of the population are indicated as effective approaches for enhancing preventive measures and fighting the infodemic responsible for the current public health crisis [16,27,28,29,30,31]. A systematic review of studies evaluating HL in relation to COVID-19, severe acute respiratory syndrome (SARS) and Middle East respiratory syndrome (MERS) was carried out by Seng et al. [32]. It appears that HL has been quite frequently addressed in the context of epidemic events. However, the relationship between the outcomes and HL has rarely been assessed. Seng et al. were able to identify 21, 17 and 32 papers addressing HL in the three epidemics of COVID-19, SARS and MERS, respectively. In most of them, the determinants of HL were assessed to be a higher level of education, older age and female gender and all were related to higher HL. 

Recognising the consequences of the infodemic accompanying the COVID-19 pandemic, the WHO started crowdsourced consultations about possible responses to the challenge. In the end, seven main approaches were identified [33]. Briefly, they include the use of evidence-based interventions and messages to support informed decisions by the public; knowledge translation to ensure that all messages are understandable and accessible; reaching out to key communities to address their concerns and information needs; establishing intersectoral partnerships to include social media, the technology sectors, academia and civil society; the development of actions based on reliable information; using the experience gained from responses to the previous epidemics and COVID-19 to develop additional management strategies to support enhanced preparedness and response [33]. 

In response to the activities of the WHO, Eysenbach proposed a model of an “information cake” in which the current infodemic is explained as a phenomenon occurring on four levels: science, policy and practice, news media and social media [34]. Using this model, he defined four pillars for infodemic management: (1) support for accurate knowledge translation, (2) knowledge refinement, filtering and fact-checking, (3) building eHealth literacy (eHL) and (4) monitoring, infodemiology, infoveillance and social listening. Eysenbach put special emphasis on eHL, that may be defined as a set of skills including searching for, finding, understanding and appraising the health information available from electronic sources [35]. It is also related to the capacity of applying the retrieved knowledge in order to respond to health challenges [35]. Still, in a broader perspective, it seems evident that during the COVID-19 pandemic, improving not only the eHL of stakeholders but also their HL should have been addressed, as has been suggested by other authors [28,36,37,38]. Possessing adequate HL is not only a tool enabling an individual to take an efficient reaction to misinformation [39], but it also ensures their social responsibility and a better understanding of measures recommended during the pandemic, e.g., social distancing [29,40]. 

The main aim of this paper was to assess the prevalence of conspiracy beliefs related to the COVID-19 pandemic in a representative sample of Poland’s Internet users. In addition, the relationships of supporting attitudes towards conspiracy theories and sociodemographic variables, HL and eHL were analysed.

## 2. Materials and Methods 

### 2.1. Survey

The analysis presented in this paper was carried out on the data originating from a survey undertaken in June 2020 on a representative sample (*n* = 1002) of Polish Internet users aged 18 and above. The computer-assisted web-based interviewing (CAWI) technique was applied. The group of respondents was recruited from a certified Internet panel by the PBS Company (Sopot, Poland), which has significant expertise in conducting opinion polls in Poland [41]. The PBS Company conforms to the Polish Programme of Interviewer Quality Control [42]. The size of the sample was determined as the minimum required after taking into consideration the size of the population (28,600,000 in 2019 according to Statistics Poland, the central statistical office in Poland [43], a fraction of 0.5 and a confidence level of 0.95. For the sample of 1002 respondents, the level of the sampling error was 3.1%. The study sample reflected the structure of the general population of adult Polish Internet users. It was standardised according to age, education (three categories), place of residence (four categories) and the Nomenclature of Territorial Units for Statistics (NUTS) 1 region. The quota was established from data reported by Statistics Poland, the main statistical office in Poland [43]. The limitations of representativeness of the samples stemming from the sampling method and the recruitment procedure were adjusted with poststratification weights obtained with the random iterative method (RIM) for the variables applied for the initial stratification and the main administrative units (voivodeships). 

The questionnaire used in the survey consisted of 55 items. It consisted of validated tools for the assessment of HL (a 16-item European Health Literacy Survey Questionnaire (HLS-EU-Q16) [44]) and eHL (an 8-item Polish version of the eHealth Literacy Scale, Pl-eHEALS) [45,46]), items asking for opinions about three popular conspiracy theories related to COVID-19 and a set of questions about the sociodemographic characteristics of respondents. Both HLS-EU-Q16 and Pl-eHEALS are based on the self-assessment of specific skills related to the ability to handle health-related information [44,45,46]. In the case of Pl-eHEALS, each respondent is asked about their ability to search, assess and use the health-related information available online [45].

The study was accepted by the Bioethical Committee of Jagiellonian University in Krakow (No 1072.6120.99.2020 of 23 April 2020). The questionnaire was completed anonymously by participants after being acquainted with the purpose of the survey and them giving their consent to join the study. 

### 2.2. Statistical Analysis

IBM SPSS v.24 software (IBM Corp., Armonk, NY, USA) was used for the statistical analysis. Absolute and relative frequencies were calculated for categorical variables, and the mean and standard deviation for continuous variables. 

A COVID-19-related Conspiracy Belief Scale (CCBS) was established on the basis of the responses to the questions asking for opinions about conspiracy theories. The response options from “I decidedly do not agree” to “I decidedly agree” were given values from “1” to “5” and totalled. The assessment of internal reliability yielded acceptable results (Cronbach’s α = 0.73, Guttman half-split coefficient = 0.69). The multivariate linear regression model was developed with the CCBS score as a dependent variable and HL, eHL and sociodemographic characteristics as independent variables. The HL and the eHL scores and age were included in the model as continuous variables. 

The total HL score was calculated only if there were at least 14 meaningful responses to the individual questions included in the HLS-EU-Q16 [44]. The response options “very difficult” and “difficult” were evaluated as “0” and “easy” and “very easy” as “1” [44]. The HL scale showed good internal consistency (Cronbach’s α = 0.90, Guttman half-split coefficient = 0.82). 

The eHL score was calculated as the sum of individual scores achieved in the Pl-eHEALS after converting the Likert scale’s responses (from “I decidedly do not agree” to “I decidedly agree”) to values of 1 to 5 [45]. The internal consistency of the Pl-eHEALS scale was also appropriate (Cronbach’s α = 0.89, Guttman half-split coefficient = 0.83). 

The sociodemographic variables used in the models, apart from age, included the level of education (four categories), the place of residence (six categories), marital status (three categories), vocational activity (five categories) and net monthly income per inhabitant of the household (four categories, including a separate category for those who refused to respond).

The predictors of three individual items which established the CCBS were determined with a multivariate logistic regression model after dichotomisation. The response options “I decidedly agree” and “I agree” were given the value “1” and the remaining options value “0”. In the multivariate logistic regression models, the same independent variables were used as in the linear regression model. 

For the independent variables used in the linear regression model, unstandardised regression coefficients (B), standard errors (SE), standardised regression coefficients (β), 95% confidence intervals (95% CI) and *p* values were provided, and the odds ratio (OR), 95% confidence intervals (95% CI) and *p* values were provided for the logistic regression. It was assumed that a *p* value < 0.05 is statistically significant. In the case of *p* < 0.10, the value was given to three decimal places, otherwise to two decimal places. 

## 3. Results

### 3.1. The Characteristics of the Study Group

The mean age of the respondents (standard deviation; SD) was 40.14 (14.21), the mean HL score was 12.87 (3.42) and eHL score 29.74 (5.14). The HL score could be calculated for only 953 respondents (95.02%) as some responses were classified as missing values. Of the study group, 50.6% were female. Persons with an upper secondary or post-secondary non-tertiary level of education comprised 48.9% of the studied group, 10.7% possessed a bachelor’s degree and 20.6% a master’s degree. In terms of location, 36.6% were inhabitants of rural areas, and 11.7% resided in large cities with a population >500,000. About 50% of the participants were married and 35% were single. In terms of employment, 47.2% were employed in the public or private sectors, 13.7% were self-employed or farmers and 10.2% were high school or university students. The number of respondents who believed that SARS-CoV-2 emerged as a result of deliberate genetic manipulations was nearly 46%. About 43% were convinced that news about the pandemic is spread to incite panic and to achieve political aims. Furthermore, 56% believed that governments are using the pandemic as a pretext for the introduction of a system for total surveillance. The characteristics of the study group are shown in Table 1. The distribution of responses to the three items asking about conspiracy beliefs is shown in the Supplementary Materials (Table S1).

### 3.2. The Assessment of Conspiracy Beliefs 

The multivariate linear regression model revealed that the CCBS score was significantly associated with age, education level, vocational status, health and eHealth literacy of respondents and their occupational status (Table 2). The CCBS was reduced by 0.04 points for each additional year of age (B = −0.04, *p* < 0.001) and by 0.07 points for a 1 point increase in HL (B = −0.07, *p* = 0.017) after controlling for other independent variables in the regression model. Those persons with the three lower levels of education returned a higher CCBS compared to those with the highest level of education (B = 0.84, *p* = 0.005; B = 0.88, *p* < 0.001 and B = 0.69, *p* = 0.041, respectively). Furthermore, students were significantly less likely to support conspiracy beliefs than persons working as employees of the private or public sector (B = −0.98, *p* = 0.01). 

Finally, a stronger belief in conspiracy theories was displayed by respondents having higher eHL. The increase in eHL by one point was associated with a 0.04 point increase in the CCBS (B = 0.04, *p* = 0.038). The beliefs score was not significantly associated with the place of residence, income level or marital status. 

The most influential individual predictors of CCBS in the sequence based on the absolute values of standardised regression coefficient were the respondents’ age (β = −0.21), education level lower than the highest level (β = 0.08 to 0.16), vocational status (β = −0.10 for the comparison of students and employees), the HL score (β = 0.08) and the eHL score (β = 0.07).

The table of correlations of the independent variables is included in the Supplementary Materials (Table S2).

### 3.3. Predictors of Specific Conspiracy Theories

The logistic regression modelling of dichotomised variables reflecting support for the three individual conspiracy theories used for the construction of the pandemic-related conspiracy belief score showed that eHL was consistently a predictor for three theories (Table 3). Persons with higher eHL were more likely to support the theory about genetic manipulations (OR, 95% CI: 1.04, 1.01–1.07), spreading panic to reach political aims (OR, 95% CI: 1.04, 1.01–1.07) and using the pandemic as a pretext for increased surveillance of the population (OR, 95% CI: 1.03, 1.01–1.06) than those with lower eHL. Higher HL was associated with lower support for the first two theories (OR, 95% CI: 0.95, 0.92–0.99, and 0.95, 0.91–0.99, respectively). 

Older respondents were less likely to agree with the last two conspiracies (OR, 95% CI: 0.96, 0.94–0.97, and 0.98, 0.97–0.99, respectively). Students were about 50% less likely to believe the theory of genetic manipulations and using panic to attain political gains than employees (OR, 95% CI: 0.44, 0.25–0.77 and 0.54, 0.31–0.93, respectively). Of the other sociodemographic variables, only marital status showed a significant association with conspiracy beliefs. Married persons were about 1.5 times more likely to believe the theory that panic is used for political purposes (OR, 95% CI: 1.49, 1.02–2.17) and those who were widowed, divorced or separated were about 1.8 times more likely to believe that the pandemic is being used as a pretext for the introduction of a system of total surveillance (OR, 95% CI: 1.77, 1.08–2.91). 

Some effects were identified with the level of education; respondents who had a master’s degree were less likely to accept the theory about the emergence of a new coronavirus being due to genetic manipulations. No significant association with the acceptance of conspiracy beliefs was found for the place of residence and the level of income per household member.

## 4. Discussion

It is perhaps surprising that between 43–56% of the adult respondents representative of the Polish population of Internet users believed the prevalent COVID-19 pandemic conspiracy theories. The CCBS score based on the responses given to three items asking about conspiracy theories was significantly associated with age, education level, vocational status, the HL and the eHL of respondents. Younger participants more than older, persons with lower education levels more than those with the highest education level and employees more than students were likely to believe in the conspiracy theories. Those with higher HL accepted such theories less frequently than those with lower HL. Contrary to expectations about the protective role of eHL, in the context of the infodemic, expressed by many authors, Polish respondents with higher eHL were more likely to believe in conspiracy theories than those with lower eHL. Of the statistically significant predictors of CCBS, the influence of eHL, based on the standardised regression coefficient, was the smallest, but it was still significant. It should be recognised that the regression model, including HL, eHL and sociodemographic factors, was able to explain only 3% of the variance of the score for conspiracy beliefs. Clearly, there must be other factors related to the support of such beliefs. 

Surveys performed in other countries have shown lower belief in conspiracy theories related to COVID-19 than in Poland. According to the study of the Pew Research Center, in the USA, 23% of adults believed that SARS-CoV-2 was deliberately developed in a laboratory [47]. This percentage was lowest for Whites (21%) but higher for Afro-Americans (26%) and Hispanics (29%). Twenty-seven percent of people aged 18–29 were believers of the theories but of those aged 65 and over, only 15% supported the concepts. 

Fifteen percent of people with a college or higher level of education believed in the theory that a new coronavirus was intentionally developed, compared to 27% of those with no more than a high school education. In the study by the Pew Research Center, the respondents were also asked about their political orientation; it was found that supporters of Republicans were more likely to believe that the new coronavirus was deliberately developed than supporters of Democrats (30% vs. 16%). 

Another study about the prevalence of the conspiracy beliefs in the USA sample was carried out by Uscinski et al. [48]. In March 2020, they asked 2000 respondents about their belief in two theories: (1) that the threat of COVID-19 was exaggerated to damage President Trump, and (2) the virus was purposefully created and spread. The survey revealed that 29% agreed with the first and 31% with the second. The strongest predictors of beliefs in these concepts were a psychological predisposition to reject expert information and accounts of major events (denialism); a psychological predisposition to see major events as a result of conspiracy theories (conspiracy thinking); political partisanship; ideological motivations; the level of religiosity and age. 

Pennycook et al. assessed misperceptions and risk perceptions about COVID-19 and intentions to change behaviour in response to the pandemic respondents in the USA (*n* = 689), the UK (*n* = 642) and Canada (*n* = 644) [49]. The surveys were carried out in late March 2020. The set of misconceptions included 22 items divided into four categories, including optimistic, pessimistic, magical and conspiratorial statements. Among the conspiratorial items, there was a statement corresponding with the theory that the coronavirus was created in a laboratory. It was supported by only 6.99% of respondents from Canada, 11.84% from the UK and 12.77% from the USA. These are much lower than in the Polish population. In general, the surveys performed by Pennycook et al. showed that nearly all other misperceptions were supported by less than 10% of respondents in all three countries. In all three samples, cognitive sophistication, indexed by analytic thinking; numeracy; basic scientific knowledge and “bullshit scepticism” were negative predictors of COVID-19 misperceptions.

In 2016, Castro-Sánchez et al. published a systematic review of papers assessing the influence of HL on infectious diseases [50]. The authors found that limited or insufficient HL was associated with a reduced acceptance of protective measures such as immunisation or an inadequate understanding of antibiotics, but this relationship was not consistent. The authors also remarked that there are significant gaps in research on HL with regard to the infectious diseases which have a high clinical and societal impact, e.g., tuberculosis or malaria. 

There are many voices postulating HL and eHL as prerequisites of an effective response to the COVID-19 pandemic [27,28,29,30,34,51]. Chong et al. [52] put a particular emphasis on the impact of eHL and health-related misinformation on the public’s adherence to the recommended measures to reduce the impact of the COVID-19 pandemic, e.g., hand washing, wearing a mask or practising physical distancing. However, to date, the association between HL, eHL and conspiracy beliefs and reactions to the COVID-19-related infodemic has rarely been addressed. Our study confirmed the relationship between a higher HL and a lower support for believing conspiracy theories related to COVID-19. However, unexpectedly, it was also found that respondents with higher eHL were more likely to believe such theories. This finding goes against the general assumptions about the potential beneficial role of eHL in handling health-related information found on the Internet. In this study, the eHEALS scale was used, which measures self-declared skills of finding, evaluating and applying electronic health information to health problems [45]. On a more general level, eHEALS reflects the respondents’ abilities and acceptance when navigating digital spaces for health-related purposes. It is also evident that the Internet has become the principal vehicle for spreading conspiracy theories and fake news [53,54]. It seems that the eHEALS score may be a measure not only of the abilities to search and use online health information but also of the openness to the information found on the Internet. The theoretical framework of eHL described by Norman and Skinner assumed that HL is one of the literacies on which eHL is based [35]. The analysis reported here showed that the potential effects of eHL and HL on conspiracy beliefs, adjusted for sociodemographic variables, are contradictory. It should be noted that in multivariate logistic models, higher eHL was a predictor of more frequent support for individual conspiracy theories. It is significant that the greatest effect on the CCBS score was confirmed for the level of education. 

Although studies reporting on the association between eHL and conspiracy belief, primarily related to COVID-19, are rarely available, many reports confirm the correlation between the degree of confidence in various types of conspiracies and the endorsement of pseudoscience with the rejection of factual science [55,56,57,58,59,60]. Recently, Čavojová et al. confirmed that scientific reasoning is negatively associated with conspiracy beliefs relevant to COVID-19 [61]. It was a stronger independent predictor of unfounded beliefs (including antivaccination attitudes) than general analytic thinking, but its role in health-related behaviours was more modest. 

The influence of conspiracy belief in the current public health emergency caused by the COVID-19 pandemic cannot be overestimated. Many authors have provided evidence of the association between conspiracy beliefs and non-adherence to the measures recommended during the pandemic. Using data from a survey of 704 respondents of about 65 nationalities, Biddlestone et al. developed a structural equation model of the intention to accept social distancing [62]. They found that beliefs in the COVID-19 conspiracy theories negatively predicted such intention both directly and indirectly, through a feeling of powerlessness. Constantinou et al. reported that beliefs in conspiracy theories are a predictor of the non-acceptance of health-related behaviours during a pandemic: refusing social distancing, pushing for mass gatherings for demonstrations and expressing a refusal to accept future vaccinations [10]. The analysis performed by Jovančević and Milićević on data coming from a survey of a convenience sample of about 400 respondents from Serbia and Latin America revealed that belief in conspiracy theories was positively associated with respect for curfews, but negatively with the recommendation of not receiving guests [63]. Imhoff and Lamberty asked respondents about belief in several conspiracy theories related to COVID-19, e.g., that a new coronavirus was intentionally presented as dangerous to mislead the public or that coronavirus was purposefully developed to reduce the population [11]. They also found that conspiracy beliefs were negatively correlated with containment-related behaviours and the perception of COVID-19 as a threat, but positively with self-centred prepping behaviour, conspiracy mentality and political orientation.

### Limitations

There are several limitations of this study which need to be indicated. Firstly, it is a cross-sectional study performed three months after the announcement of an epidemic state in Poland. Therefore, the potential evaluation of the beliefs in conspiracy theories could not be addressed. However, it would be interesting to ascertain if the conspiracy beliefs circulating before the first cases of COVID-19 were confirmed in Poland were supported by as many people as was found in the survey carried out three months later. 

The measure of conspiracy beliefs related to COVID-19 was constructed based on the opinions expressed by respondents about only three conspiracy theories. During the COVID-19 pandemic, many other conspiracy theories have emerged and been disseminated on the Internet and other media. As this survey was designed to cover other aspects related to the pandemic, e.g., the use of eHealth services, the acceptance of preventive measures and feelings about the future (which are not included in this paper), it was decided that only three conspiracy theories were to be addressed in the questionnaire. Furthermore, the decision to include particular theories was based on an arbitrary decision and a subjective assessment of issues gaining the greatest popularity among the public. In parallel, extreme ideas, like a complete denial of the pandemic or that 5G technology is used to spread the new coronavirus, were avoided. On the one hand, in this study, the decisions resulted in a relatively large proportion of supportive opinions for conspiracy statements. It is not clear if the unexpected positive relationship between higher eHL and the support for conspiracy beliefs would also apply to extreme conspiracy ideas. 

In the previous study [64], the group of respondents for whom the HL score could not be calculated in line with the recommendations of the European Health Literacy Survey project for the 16-item EHLS questionnaire was much larger [44]. Still, there was about 5% of such respondents. 

It should also be stressed that the survey was performed online, and the analysis could not include non-users of the Internet. According to the data from Statistics Poland, 13.3% of households do not have access to the Internet [43]. A report published in 2019 stated that 100% of those aged 18–24 accessed the Internet at least once a week, as did 99% of those aged 24–34 [65]. For people aged 55–64, the percentage was 56% and for those 65 years old and over, the percentage fell to 26%. Consequently, younger users were overrepresented in the study sample as there is still a significant digital divide for the older population. The role of the Internet as a vehicle for spreading conspiracy theories and fake news has been studied and confirmed in many reports. It would be interesting to investigate if individuals without Internet access, or not using it for other reasons, are as prone to believe conspiracy theories related to the pandemic. 

## 5. Conclusions

The analysis reported in this paper showed that age, level of education and vocational status are the strongest predictors of conspiracy beliefs and that HL and eHL also play a role. Considering eHL, it was found that a higher level of eHL is associated with higher likelihood of support for conspiracy beliefs. A increase by 1 point of the eHL score was associated with about a 4% increase in support for the individual conspiracy theories assessed in this survey. Although the effect is not large, the finding is counterintuitive and opposite to the general expectation that higher eHL should support the resilience of individuals exposed to the infodemic accompanying the COVID-19 pandemic. It should be noted that the overall effect of HL, eHL and sociodemographic factors assessed with the multivariate regression model was rather low. The low observed effects of HL and eHL could be associated with the use of measuring instruments based on the respondent’s self-assessment. In future studies, the use of tools assessing objective skills should be considered. Supposedly, other factors may have an important role in shaping attitudes toward conspiracy theories. Nonetheless, it is a unique Polish study, which has explored the relationship between HL and eHL and the conspiracy beliefs related to the COVID-19 pandemic. The level of influence found for HL may be disappointing, and that for eHL is disturbing, however, these findings suggest that there should be a search for other factors on which to focus, which may be significant in combatting the current infodemic. Further studies should also investigate the associations between HL and eHL and health-related behaviours, adherence to the recommendations for reducing the risk of contracting coronavirus infection and the perception of misinformation and fake news flooding the Internet and social media. A more detailed assessment of the relationship between media literacy and eHL is also recommended.

## Figures and Tables

**Table 1:** Characteristics of the study group.

Variable	Response Categories	Respondents % (*n*)
Gender	women	50.6 (507)
	men	49.4 (495)
Education level	lower than upper secondary	19.8 (199)
	upper secondary or post-secondary non-tertiary	48.9 (490)
	bachelor’s degree	10.7 (107)
	masters’ degree or higher	20.6 (206)
Place of residence	rural	36.6 (366)
	urban < 20,000	10.9 (110)
	urban from 20,000 to <100,000	21.0 (211)
	urban from 100,000 to <200,000	9.1 (92)
	urban from 200,000 to <500,000	10.6 (107)
	urban from 500,000	11.7 (117)
Marital status	single	34.5 (345)
	married	50.8 (509)
	widowed or divorced or separated	14.7 (147)
Vocational status	employee	47.2 (473)
	self-employed or farmer	13.7 (138)
	on a disability pension or retired	9.6 (96)
	university or school student	10.2 (102)
	vocationally inactive including unemployed	19.3 (194)
Net monthly income per household inhabitant	≤PLN 1500 *	26.4 (265)
	>PLN 1500–3000	42.6 (427)
	>PLN 3000	18.0 (180)
	refused to disclose	13.0 (130)
Coronavirus responsible for the COVID-19 pandemic is a result of genetic manipulations carried out by man.	I decidedly do not agree	8.5 (85)
	I do not agree	9.7 (97)
	difficult to say	36.0 (361)
	I agree	27.1 (272)
	I decidedly agree	18.7 (187)
The coronavirus news is made up to spread panic and to achieve a political aim.	I decidedly do not agree	10.2 (102)
	I do not agree	15.5 (155)
	difficult to say	32.6 (327)
	I agree	23.1 (231)
	I decidedly agree	18.7 (187)
Governments treat the COVID-19 pandemic as a pretext for the introduction of total surveillance of the population	I decidedly do not agree	5.2 (52)
	I do not agree	8.6 (86)
	difficult to say	30.1 (302)
	I agree	32.7 (328)
	I decidedly agree	23.4 (234)

* PLN—current ISO4217 code for Polish zloty.

**Table 2:** Multivariate linear regression model for predictors of the COVID-19 Conspiracy Belief Score.

Independent Variables	Categories of an Independent Variable	COVID-19 Conspiracy Belief Score (CCBS)				
		Mean (SD)	B (SE)	β	95%CI	*p* ^&^
Health literacy			−0.067 (0.03)	−0.08	−0.12 to −0.01	0.017
eHealth literacy			0.039 (0.02)	0.07	0.002 to 0.08	0.038
Age			−0.04 (0.01)	−0.21	−0.06 to −0.02	<0.001
Gender	women ^#^	10.34 (2.63)	ref.			
	men	10.15 (2.92)	−0.151 (0.18)	−0.03	−0.51 to 0.21	0.41
Place of residence	rural ^#^	10.25 (2.79)	ref.			
	urban < 20,000	10.37 (3.01)	0.167 (0.31)	0.02	−0.43 to 0.77	0.58
	urban from 20,000 to <100,000	10.45 (2.67)	0.212 (0.25)	0.03	−0.27 to 0.7	0.39
	urban from 100,000 to <200,000	10.28 (2.29)	0.114 (0.33)	0.01	−0.53 to 0.76	0.73
	urban from 200,000 to <500,000	10.28 (2.71)	0.151 (0.31)	0.02	−0.46 to 0.76	0.63
	urban from 500,000	9.72 (3.08)	−0.264 (0.3)	−0.03	−0.86 to 0.33	0.38
Education level	lower than upper secondary	10.48 (2.71)	0.838 (0.3)	0.12	0.25 to 1.42	0.005
	upper secondary or post-secondary non-tertiary	10.36 (2.79)	0.878 (0.24)	0.16	0.4 to 1.36	<0.001
	bachelor’s degree	10.36 (2.48)	0.693 (0.34)	0.08	0.03 to 1.36	0.041
	masters’ degree or higher ^#^	9.69 (2.90)	ref.			
Marital status	single	10.21 (2.82)	−0.482 (0.25)	−0.08	−0.97 to 0.01	0.055
	married ^#^	10.25 (2.71)	ref.			
	widowed, divorced or separated	10.31 (2.90)	0.046 (0.27)	0.01	−0.49 to 0.58	0.87
Vocational status	employee ^#^	10.36 (2.82)	ref.			
	self-employed or farmer	10.22 (2.78)	0.069 (0.28)	0.01	−0.48 to 0.61	0.80
	on disability pension or retired	9.82 (3.00)	0.143 (0.38)	0.02	−0.59 to 0.88	0.70
	university or school student	9.85 (2.69)	−0.978 (0.37)	−0.10	−1.7 to −0.26	0.01
	vocationally inactive	10.40 (2.56)	−0.071 (0.25)	−0.01	−0.56 to 0.42	0.78
Net income per household member	≤PLN 1500 *^,#^	10.51 (2.68)	ref.			
	>PLN 1500–3000	10.13 (2.73)	−0.262 (0.23)	−0.05	−0.7 to 0.18	0.25
	>PLN 3000	10.24 (2.94)	−0.151 (0.28)	−0.02	−0.71 to 0.41	0.55
	refused to disclose	10.09 (2.88)	−0.186 (0.31)	−0.02	−0.8 to 0.42	0.55

R^2^ = 0.05, adjusted R^2^ = 0.03, F = 2434, *p* < 0.001, abbreviations: SD—standard deviation, B—unstandardised regression coefficient; SE—standard error; β—standardised regression coefficient; ref.—reference category of independent variable; ^&^ *p* value for multivariate linear regression, ^#^ reference category of the independent variable in the linear regression model; * PLN—current ISO4217 code for Polish zloty (PLN 1 = EUR 0.2246 according to the exchange rate on 15 June 2020).

**Table 3:** Logistic regression models for dichotomised variables reflecting views on individual conspiracy theories included in the questionnaire.

Independent Variables	Categories of an Independent Variable	Genetic Manipulations			Panic and Political Purposes			The Introduction of Invigilation		
		OR	95%CI	*p* ^&^	OR	95%CI	*p* ^&^	OR	95%CI	*p* ^&^
Health literacy		0.95	0.92–1.00	0.028	0.95	0.91–0.99	0.021	0.97	0.93–1.01	0.13
eHealth literacy		1.04	1.01–1.07	0.005	1.04	1.01–1.07	0.006	1.03	1.01–1.06	0.016
Age		0.99	0.98–1.01	0.44	0.96	0.94–0.97	<0.001	0.98	0.97–0.99	0.005
Gender	women vs. men	1.00	0.76–1.31	0.98	0.89	0.68–1.18	0.42	1.18	0.9–1.55	0.24
Place of residence	rural ^#^									
	urban < 20,000	1.37	0.88–2.15	0.17	0.98	0.62–1.55	0.93	1.17	0.74–1.83	0.50
	urban from 20,000 to <100,000	0.82	0.57–1.19	0.30	1.15	0.8–1.67	0.45	1.33	0.92–1.92	0.13
	urban from 100,000 to <200,000	0.84	0.52–1.36	0.48	1.03	0.62–1.69	0.92	1.25	0.77–2.04	0.36
	urban from 200,000 to <500,000	0.8	0.51–1.27	0.34	1.44	0.91–2.29	0.12	1.04	0.66–1.64	0.87
	urban from 500,000	0.71	0.45–1.11	0.13	1.1	0.7–1.73	0.69	1.21	0.77–1.89	0.40
Net income per household member	≤PLN 1500 *^,#^									
	>PLN 1500–3000	0.86	0.61–1.19	0.36	0.99	0.71–1.39	0.96	0.89	0.64–1.25	0.51
	>PLN 3000	0.86	0.57–1.31	0.49	1.35	0.89–2.07	0.16	0.92	0.61–1.41	0.71
	refused to disclose	1.04	0.66–1.65	0.86	0.90	0.57–1.44	0.67	0.90	0.57–1.42	0.64
Education level	lower than upper secondary ^#^									
	upper secondary or post-secondary non-tertiary	0.99	0.7–1.42	0.97	1.12	0.78–1.6	0.55	1.12	0.79–1.6	0.53
	bachelor’s degree	0.92	0.56–1.52	0.75	0.99	0.59–1.66	0.98	1.49	0.89–2.48	0.13
	masters’ degree or higher	0.5	0.32–0.78	0.002	0.68	0.43–1.07	0.093	0.98	0.63–1.52	0.93
Marital status	single ^#^									
	married	1.21	0.84–1.75	0.31	1.49	1.02–2.17	0.038	1.23	0.85–1.78	0.27
	widowed, divorced or separated	1.05	0.64–1.72	0.84	1.43	0.87–2.36	0.16	1.77	1.08–2.91	0.023
Vocational status	employee ^#^									
	self-employed or farmer	0.99	0.66–1.49	0.95	1.08	0.71–1.64	0.71	0.97	0.65–1.47	0.90
	on disability pension or retired	0.71	0.41–1.25	0.24	1.77	0.99–3.14	0.052	0.84	0.48–1.46	0.54
	university or school student	0.44	0.25–0.77	0.004	0.54	0.31–0.93	0.028	0.77	0.45–1.33	0.35
	vocationally inactive	0.8	0.55–1.16	0.23	0.95	0.65–1.38	0.77	0.89	0.62–1.29	0.55

Regression models for: (1) theory of coronavirus being a result of genetic manipulations in a laboratory: Hosmer–Lemeshow chi^2^ test = 9,14, df = 8, *p* = 0.33, Nagelkerke R^2^ = 0.06; (2) theory of deliberate spreading of the panic for a political aim: Hosmer–Lemeshow chi^2^ test = 6.14, df = 8, *p* = 0.63, Nagelkerke R^2^ = 0.08; (3) the introduction of total surveillance after pandemic: Hosmer–Lemeshow chi^2^ test = 11.01, df = 8, *p* = 0.20, Nagelkerke R^2^ = 0.04; ^&^ *p* value for multivariate logistic regression, ^#^ reference category of the independent variable in the logistic regression model, * PLN—current ISO4217 code for Polish zloty.

## Supplementary Materials

The following are available online at https://www.mdpi.com/1660-4601/17/21/7818/s1, Table S1: The distribution of responses to items asking about conspiracy beliefs related to COVID-19, Table S2: Spearman correlations between independent variables used in regression models.
