# Supplementary material for: The Determinants of Conspiracy Beliefs Related to the COVID-19 Pandemic in a Nationally Representative Sample of Internet Users
Source: Int J Environ Res Public Health. 2020 Oct 26;17(21):7818. doi: 10.3390/ijerph17217818 (PMC7663717; doi:10.3390/ijerph17217818)
Supplement: Supplementary file 1 [file ijerph-17-07818-s001.pdf]

Supplementary Materials

Table S1. The distribution of responses to items asking about conspiracy beliefs related to COVID-19.

Items related to conspiracy beliefs	I decidedly do not agree % (n)	I do not agree % (n)	difficult to say % (n)	I agree % (n)	I decidedly agree % (n)
Coronavirus responsible for the COVID-19 pandemic is a result of genetic manipulations carried out by man.	8.48 (85)	9.68 (97)	36.03 (361)	27.15 (272)	18.66 (187)
The coronavirus news is made up to spread panic and to achieve a political aim.	10.18 (102)	15.47 (155)	32.63 (327)	23.05 (231)	18.66 (187)
Governments treat a COVID-19 pandemic as a pretext for the introduction of total surveillance of the population.	5.19 (52)	8.58 (86)	30.14 (302)	32.73 (328)	23.35 (234)

Table S2. Spearman correlations for independent variables used in regression models.

	1	2	3	4	5	6	7	8
1. Health literacy	1							
2. eHealth literacy	0.403**	1						
3. Age	0.169**	0.086**	1					
4. Gender	-0.01	-0.006	0.011	1				
5. Place of residence	-0.017	-0.004	0.051	-0.025	1			
6. Education level	-0.047	0.023	0.126**	-0.039	0.011	1		
7. Marital status	0.079*	0.049	0.592**	-0.124**	-0.01	0.081**	1	
8. Vocational status	-0.004	-0.064*	-0.083**	-0.076*	-0.073*	-0.173**	-0.114**	1
9. Net income	-0.045	-0.023	-0.028	0.090**	0.101**	0.113**	-0.036	-0.067*

* $p < 0.05$, ** $p < 0.01$.
